# Institutional Needs

**Published:** 1920-06-19

**Authors:** 


### Institutional Needs.

JOHN BOND'S CRYSTAL PALACE.
MARKING INK.

Bond's marking ink was the first in the field, and its name evokes childish memories of the fascinating little old stall at the Crystal Palace in its palmy days. It has held its own ever since in the teeth of countless competitors, and its qualities were never finer than at the present time. It does not blot or dry on the pen while in use. It glides swiftly and neatly over the fabric and lends itself particularly well to the pen-painting process necessary for bold marking. It does not become effaced even after long usage, and the non-heat variety shortens the labours of the operator. It remains to be said that the price has not appreciably altered; at any rate the 1s. bottle is still about the same size as it used to be.

